# Supplementary material for: The Evolution and Characterization of the RNA Interference Pathways in Lophotrochozoa
Source: Genome Biol Evol. 2024 May 7;16(5):evae098. doi: 10.1093/gbe/evae098 (PMC11114477; doi:10.1093/gbe/evae098)
Supplement: evae098_Supplementary_Data [file evae098_supplementary_data.zip › Supplementary_materials.zip!/selected_miRNAs/NoGe_selected_miRNAs/mirdeep_pdf_files/NMRB01000152.1_Notospermus_geniculatus_strain_Ushimado_scaffold152,_whole_genome_shotgun_sequence_5839.pdf]

Provisional ID	:	NMRB01000152.1_Notospermus_geniculatus_strain_Ushimado_scaffold152,_whole_genome_shotgun_sequence_5839
Score total	:	17140
Score for star read(s)	:	3.9
Score for read counts	:	17130.8
Score for mfe	:	2.3
Score for randfold	:	0
Score for cons. seed	:	3
Total read count	:	33613
Mature read count	:	33528
Loop read count	:	0
Star read count	:	85

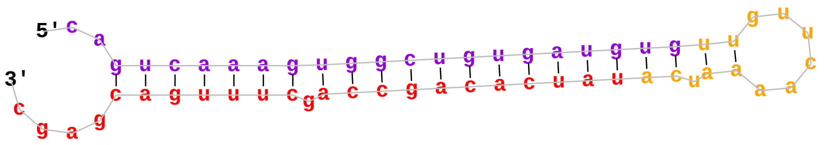

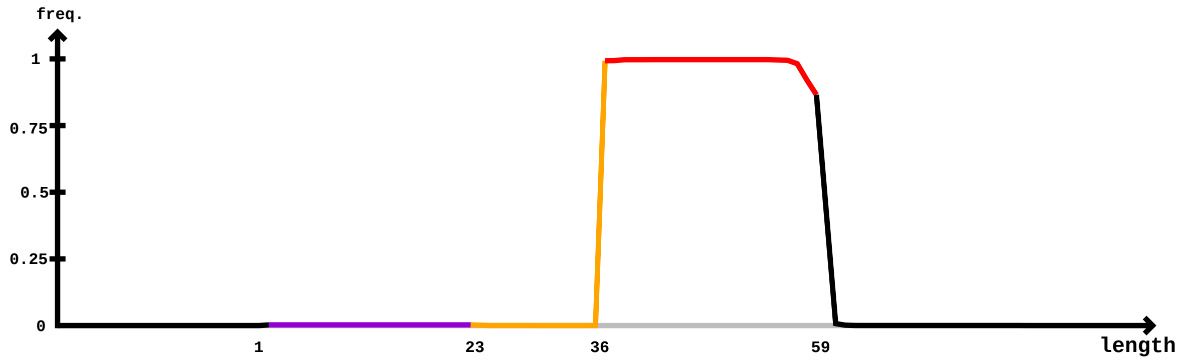

Star **Mature**

```
5'- gaaguuggaugcccccagagcagucaaaguggcugugauguguuguucaaaaaucauaucacagccagcuuugacgagcuuuguggguguugucgcuuucauugcguaaggca  -3'   obs
    gaaguuggaugcccccagagcagucaaaguggcugugauguguuguucaaaaaucauaucacagccagcuuugacgagcuuuguggguguugucgcuuucauugcguaaggca        exp
    (((((..(((((((.((((((.((((((((((((((((((((((......)).))))))))))))).)))))))..)))))).)))))))...)))))...(((.....)))  reads   mm   sample
    ....................cagucaaaguggcugugaugug.......................................................................  11      0    Mm3
    ....................cagucaaaguggcugugaugugu......................................................................  1       0    Mm3
    ....................cagucaaaguggcugugauguguuguuc.................................................................  1       0    Mm3
    ....................cagucaaaguggcugugauguguuguucaa...............................................................  1       0    Mm3
    .......................................................Cuaucacagccagcuuugacgagcu.................................  2       1    Mm3
    ........................................................uaucacagccagcuuugU.......................................  1       1    Mm3
    ........................................................uaucacagccagcuuuga.......................................  7       0    Mm3
    ........................................................uaucacGgccagcuuuga.......................................  1       1    Mm3
    ........................................................uaucacagccagcuuugac......................................  4       0    Mm3
    ........................................................uaucacagccagcuuugaA......................................  2       1    Mm3
    ........................................................uaucacagccagcuuugacA.....................................  1       1    Mm3
    ........................................................Caucacagccagcuuugacg.....................................  1       1    Mm3
    ........................................................uaucacagccagcuuugacg.....................................  54      0    Mm3
    ........................................................uaucacagUcagcuuugacg.....................................  1       1    Mm3
    ........................................................uGucacagccagcuuugacg.....................................  1       1    Mm3
    ........................................................uaucacagccagcuuugacU.....................................  2       1    Mm3
    ........................................................uaucacagccagcuuugacga....................................  80      0    Mm3
    ........................................................uaucacagccagcuuugacgG....................................  1       1    Mm3
    ........................................................uaucacagccagcuuugacgaU...................................  8       1    Mm3
    ........................................................uaucacagccagcuuuUacgag...................................  1       1    Mm3
    ........................................................uaucacagccagUuuugacgag...................................  1       1    Mm3
    ........................................................uaucacagUcagcuuugacgag...................................  1       1    Mm3
    ........................................................uaucacaUccagcuuugacgag...................................  1       1    Mm3
    ........................................................uaucacagccGgcuuugacgag...................................  1       1    Mm3
    ........................................................uaucacagccagcuuugacgag...................................  220     0    Mm3
    ........................................................uaucacagccagcuuuAacgag...................................  1       1    Mm3
    ........................................................uaucacagccagcuuuUacgagc..................................  2       1    Mm3
    ........................................................uaucacagccagcuuuCacgagc..................................  1       1    Mm3
    ........................................................uaucacagccagcuuugGcgagc..................................  2       1    Mm3
    ........................................................uaucacagccaUcuuugacgagc..................................  1       1    Mm3
    ........................................................uaucacagccagcuuugacAagc..................................  1       1    Mm3
    ........................................................uaucacagccagcuuugCcgagc..................................  8       1    Mm3
    ........................................................uaucacagccUgcuuugacgagc..................................  3       1    Mm3
```

Star **Mature**

```
gaaguuggaugcccccagagcagucaaaguggcugugauguguuguucaaaaucauaucacagccagcuuugacgagcuuuguggguguugucgcuuucauugcguaaggca
.......................................................uaucacagccagcuuugacgaCc..................................   1     1   Mm3
.......................................................uaucacaUccagcuuugacgagc..................................   1     1   Mm3
.......................................................uaucacagccagcuuugacgagc..................................   1415  0   Mm3
.......................................................uaucacagccagcCuugacgagc..................................   1     1   Mm3
.......................................................uaucacagccagcuuugacgagG..................................   6     1   Mm3
.......................................................uUucacagccagcuuugacgagc..................................   2     1   Mm3
.......................................................uaucacagcAagcuuugacgagc..................................   1     1   Mm3
.......................................................uaucacagccagcuuugUcgagc..................................   8     1   Mm3
.......................................................uaucacUgccagcuuugacgagc..................................   1     1   Mm3
.......................................................uaucacagccagcGuugacgagc..................................   1     1   Mm3
.......................................................uaucacagccagcuCugacgagc..................................   1     1   Mm3
.......................................................uaucacagUcagcuuugacgagc..................................   1     1   Mm3
.......................................................uaucacagccCgcuuugacgagc..................................   1     1   Mm3
.......................................................uaucacagcUagcuuugacgagc..................................   2     1   Mm3
.......................................................uaucacagccGgcuuugacgagc..................................   1     1   Mm3
.......................................................uaucacagccagUuuugacgagc..................................   1     1   Mm3
.......................................................uaucacagccagcuuuAacgagc..................................   6     1   Mm3
.......................................................uaucacagccagcuuugacgUgc..................................   1     1   Mm3
.......................................................uaucacagccagcuuugacgagA..................................   41    1   Mm3
.......................................................uaucacagAcagcuuugacgagc..................................   1     1   Mm3
.......................................................uaucacagccagcuuugacgagU..................................   102   1   Mm3
.......................................................uaAcacagccagcuuugacgagcu.................................   2     1   Mm3
.......................................................uaucacagccagcuuugUcgagcu.................................   10    1   Mm3
.......................................................uaucaAagccagcuuugacgagcu.................................   1     1   Mm3
.......................................................uaucaUagccagcuuugacgagcu.................................   1     1   Mm3
.......................................................uaCcacagccagcuuugacgagcu.................................   1     1   Mm3
.......................................................uaucacGgccagcuuugacgagcu.................................   3     1   Mm3
.......................................................uaucacagccagcuuugGcgagcu.................................   3     1   Mm3
.......................................................uaucacagccagcuuugacUagcu.................................   1     1   Mm3
.......................................................uaucacagccagcuuuAacgagcu.................................   5     1   Mm3
.......................................................uaucacagccagcuuugCcgagcu.................................   8     1   Mm3
.......................................................uaucacagccCgcuuugacgagcu.................................   3     1   Mm3
.......................................................uaucacagcUagcuuugacgagcu.................................   1     1   Mm3
.......................................................Naucacagccagcuuugacgagcu.................................   3     1   Mm3
.......................................................uaucacagccaUcuuugacgagcu.................................   1     1   Mm3
.......................................................uaucacagccGgcuuugacgagcu.................................   3     1   Mm3
.......................................................uaucacagccagcuuugacgagcu.................................   2082  0   Mm3
.......................................................uaucacagccagcuuugacgagUu.................................   5     1   Mm3
.......................................................uaucacagccUgcuuugacgagcu.................................   3     1   Mm3
.......................................................uaucacagccagcuuuUacgagcu.................................   4     1   Mm3
.......................................................Aaucacagccagcuuugacgagcu.................................   1     1   Mm3
.......................................................uaucacagccagcCuugacgagcu.................................   2     1   Mm3
.......................................................uaucacNgccagcuuugacgagcu.................................   1     1   Mm3
.......................................................uaucacagUcagcuuugacgagcu.................................   2     1   Mm3
.......................................................uaucacagccagcuuuCacgagcu.................................   8     1   Mm3
.......................................................uaucacagccagcuuugacgagcG.................................   43    1   Mm3
.......................................................uaucacaCccagcuuugacgagcu.................................   1     1   Mm3
.......................................................uaucacagccagcuuugacgagcC.................................   7     1   Mm3
.......................................................uaucacagccagcuuugacgagcA.................................   14    1   Mm3
.......................................................uaucacagccagcuuugacgagcuC................................   2     1   Mm3
.......................................................uaucacagccagcuuugacgagcuu................................   42    0   Mm3
.......................................................uaucacagccagcuuugCcgagcuu................................   1     1   Mm3
.......................................................uaucacagccagcuuAgacgagcuu................................   1     1   Mm3
.......................................................uaucacagccagcuuugacgagcuG................................   3     1   Mm3
.......................................................uaucacagccagcuuugacgagcuA................................   2     1   Mm3
.......................................................uaucacagccagcuuugacgagcuuu...............................   3     0   Mm3
.......................................................uaucacagccagcuuugGcgagcuuu...............................   1     1   Mm3
.......................................................uaucacagccagcuuugaUgagcuuu...............................   3     1   Mm3
.......................................................uaGcacagccagcuuugacgagcuuu...............................   1     1   Mm3
.......................................................uaucacagccagcuuugacgagcuuuU..............................   1     1   Mm3
........................................................aucacagccagcuuugacgagcu.................................   2     0   Mm3
........................................................aucacagccagcuuugacgagcG.................................   1     1   Mm3
........................................................aucacagccagcuuugaUgagcuuu...............................   1     1   Mm3
.........................................................ucacagccagcuuugacga....................................   1     0   Mm3
.........................................................ucacagccagcuuugacgagc..................................   2     0   Mm3
.........................................................ucacagccagcuuugaGgagc..................................   1     1   Mm3
.........................................................ucacagccagcuuugacgagcC.................................   5     1   Mm3
..........................................................cacagccagcuuugacgagc..................................   2     0   Mm3
..........................................................cacagccagcuuugacgagcu.................................   1     0   Mm3
...........................................................acagccagcuuugacgCgcu.................................   1     1   Mm3
```

```
                    Star                              Mature
gaaguuggaugcccccagagcagucaaaguggcugugauguguuguucaaaaucauaucacagccagcuuugacgagcuuugugggguguugucgcuuucauugcguaaggca
............................................................cagccagcuuugacgagc...................................   1      0      Mm3

....................cagucaaaguggcugugaugug.......................................................................   30     0      Fm3
....................cagucaaaguggcugugCugug.......................................................................   1      1      Fm3
......................................................Cuaucacagccagcuuugacga.....................................   4      1      Fm3
.......................................................uaucacagccagcuuuga........................................   10     0      Fm3
.......................................................uaucacagccagcuuugU........................................   1      1      Fm3
.......................................................uaucacagccagcuuugaA.......................................   4      1      Fm3
.......................................................uaucacagUcagcuuugacg......................................   10     1      Fm3
.......................................................uaucacagccagcCuugacg......................................   4      1      Fm3
.......................................................uaucacagccagcuuugacA......................................   3      1      Fm3
.......................................................uaucacagccagcuuugacU......................................   7      1      Fm3
.......................................................uaucacagccUgcuuugacg......................................   1      1      Fm3
.......................................................uaucacagccagcuuugUcg......................................   1      1      Fm3
.......................................................uaucacagccagcuuugacg......................................   190    0      Fm3
.......................................................uaucacCgccagcuuugacga.....................................   1      1      Fm3
.......................................................uaucacagccUgcuuugacga.....................................   2      1      Fm3
.......................................................uaucacagccagcCuugacga.....................................   10     1      Fm3
.......................................................uaucacagcUagcuuugacga.....................................   1      1      Fm3
.......................................................uaucacagccagcuuuCacga.....................................   2      1      Fm3
.......................................................uaucacagccagcuuugCcga.....................................   1      1      Fm3
.......................................................uaucacagccagcuuugacgU.....................................   1      1      Fm3
.......................................................uaucaGagccagcuuugacga.....................................   1      1      Fm3
.......................................................uaucacagccagcuuugacga.....................................   682    0      Fm3
.......................................................uaucacagccGgcuuugacga.....................................   1      1      Fm3
.......................................................uaucacagccagcuuuAacga.....................................   2      1      Fm3
.......................................................uGucacagccagcuuugacga.....................................   1      1      Fm3
.......................................................uaucacagccagcuuuUacga.....................................   2      1      Fm3
.......................................................uaucacagccagcuuugGcga.....................................   1      1      Fm3
.......................................................uaucacagccCgcuuugacga.....................................   1      1      Fm3
.......................................................uaucacagccagcuuugacgaA....................................   4      1      Fm3
.......................................................uaucacagccagcuuuAacgag....................................   1      1      Fm3
.......................................................uaucacUgccagcuuugacgag....................................   1      1      Fm3
.......................................................uaucacagcUagcuuugacgag....................................   4      1      Fm3
.......................................................uauUacagccagcuuugacgag....................................   1      1      Fm3
.......................................................uaucacagccagcuuugGcgag....................................   1      1      Fm3
.......................................................Caucacagccagcuuugacgag....................................   1      1      Fm3
.......................................................uaucacagccagcuuugacgag....................................   729    0      Fm3
.......................................................uaucacaUccagcuuugacgag....................................   1      1      Fm3
.......................................................uaucacagccagcuuugCcgag....................................   1      1      Fm3
.......................................................uaucacagccCgcuuugacgag....................................   1      1      Fm3
.......................................................uaucacagccagcuuugacgaU....................................   21     1      Fm3
.......................................................uaucUcagccagcuuugacgag....................................   1      1      Fm3
.......................................................uaucacagccagcuuuCacgag....................................   1      1      Fm3
.......................................................uaucacagccagcuuugUcgag....................................   3      1      Fm3
.......................................................uaucacagccagcuuugacAag....................................   1      1      Fm3
.......................................................uaucacagccagcuuugaAgag....................................   1      1      Fm3
.......................................................uaucacagcGagcuuugacgag....................................   1      1      Fm3
.......................................................uaucacagccagcCuugacgag....................................   3      1      Fm3
.......................................................uaucacagccagcuuugacgagG...................................   4      1      Fm3
.......................................................uaucacagccagcuuugacgaCc...................................   1      1      Fm3
.......................................................Aaucacagccagcuuugacgagc...................................   1      1      Fm3
.......................................................uaucacagccagcuuugacgUgc...................................   1      1      Fm3
.......................................................uaucacagccagcuuuUacgagc...................................   4      1      Fm3
.......................................................uaucacagccGgcuuugacgagc...................................   5      1      Fm3
.......................................................uaucacagcGagcuuugacgagc...................................   1      1      Fm3
.......................................................uauGacagccagcuuugacgagc...................................   1      1      Fm3
.......................................................uaucacagccagcuuugUcgagc...................................   3      1      Fm3
.......................................................uaucacagccagcuuugacgagU...................................   224    1      Fm3
.......................................................uaucacagcAagcuuugacgagc...................................   2      1      Fm3
.......................................................uaucacagccagUuuugacgagc...................................   2      1      Fm3
.......................................................uaucacagccagcuuugCcgagc...................................   5      1      Fm3
.......................................................uaucGcagccagcuuugacgagc...................................   1      1      Fm3
.......................................................uaucacagccagcuuugacgagc...................................   2354   0      Fm3
.......................................................uaucacagccagcuuugacgagA...................................   209    1      Fm3
.......................................................uaucacagccCgcuuugacgagc...................................   2      1      Fm3
.......................................................uauUacagccagcuuugacgagc...................................   2      1      Fm3
.......................................................uaucacagccagcuuugGcgagc...................................   7      1      Fm3
.......................................................uaucacagccagcuuugacAagc...................................   1      1      Fm3
.......................................................uaucacagccagAuuugacgagc...................................   1      1      Fm3
```

```
                    Star                                 Mature
gaaguuggaugcccccagagcagucaaaguggcugugauguguuguucaaaaucauaucacagccagcuuugacgagcuuuguggguguugucgcuuucauugcguaaggca
.......................................................uauAacagccagcuuugacgagc..................................   1      1    Fm3
.......................................................uaucacagccagcuuuAacgagc..................................   3      1    Fm3
.......................................................uaucacagccagcuuuCacgagc..................................   2      1    Fm3
.......................................................uaucacagccUgcuuugacgagc..................................   22     1    Fm3
.......................................................uaucacagcUagcuuugacgagc..................................   9      1    Fm3
.......................................................uaucacagUcagcuuugacgagc..................................   3      1    Fm3
.......................................................uaucacagccagcuuugUcgagcu.................................   9      1    Fm3
.......................................................uaucacagccagcuuugacgagAu.................................   1      1    Fm3
.......................................................uaucacGgccagcuuugacgagcu.................................   1      1    Fm3
.......................................................uUucacagccagcuuugacgagcu.................................   1      1    Fm3
.......................................................uaucacagccagcCuugacgagcu.................................   6      1    Fm3
.......................................................uaucacagcUagcuuugacgagcu.................................   7      1    Fm3
.......................................................uaucacagccagcuuuCacgagcu.................................   1      1    Fm3
.......................................................uaucacUgccagcuuugacgagcu.................................   1      1    Fm3
.......................................................uaucacaUccagcuuugacgagcu.................................   4      1    Fm3
.......................................................uaucacagccagcuuugacUagcu.................................   1      1    Fm3
.......................................................uaucacagccagcuuuUacgagcu.................................   4      1    Fm3
.......................................................uaucacagccagcuuugacgagcC.................................   1      1    Fm3
.......................................................uaucacagccGgcuuugacgagcu.................................   1      1    Fm3
.......................................................uaucacagccagcuuuAacgagcu.................................   3      1    Fm3
.......................................................uaGcacagccagcuuugacgagcu.................................   2      1    Fm3
.......................................................uaucacagccagcuuugacgagcu.................................   1401   0    Fm3
.......................................................uaucacagccagcuuugCcgagcu.................................   3      1    Fm3
.......................................................uaucacagUcagcuuugacgagcu.................................   1      1    Fm3
.......................................................Naucacagccagcuuugacgagcu.................................   1      1    Fm3
.......................................................uaucacagccagcuuugacgagUu.................................   3      1    Fm3
.......................................................uaucacagccagcuuugGcgagcu.................................   2      1    Fm3
.......................................................uaucacagccagcuuugacgagcA.................................   10     1    Fm3
.......................................................uaucacagccagUuuugacgagcu.................................   1      1    Fm3
.......................................................uaucacagccagcuuugacgagcCu................................   1      1    Fm3
.......................................................uaucacagccagcuuugacgagcuA................................   3      1    Fm3
........................................................aucacagccagcuuugacgagcu.................................   4      0    Fm3
.........................................................ucacagccagcuuugaAgagc..................................   2      1    Fm3
.........................................................ucacagccagcuuugacgagc..................................   4      0    Fm3
.........................................................ucacagccagcuuugaGgagc..................................   1      1    Fm3
.........................................................ucacagccagcuuugacgagcC.................................   2      1    Fm3
.........................................................ucacagccagcuuugacgagcu.................................   2      0    Fm3

....................cagucaaaguggcugugaugug......................................................................   7      0    Mm2
......................................................Cuaucacagccagcuuugacga....................................   1      1    Mm2
.......................................................uaucacagccagcuuuga.......................................   8      0    Mm2
.......................................................uaucacagccagcuuugaA......................................   5      1    Mm2
.......................................................uaucacagccagcuuugacg.....................................   31     0    Mm2
.......................................................uaucacagccagUuuugacg.....................................   1      1    Mm2
.......................................................uaucacagccagcuuugacA.....................................   1      1    Mm2
.......................................................uaucacagccagcuuugacU.....................................   1      1    Mm2
.......................................................uaucaAagccagcuuugacga....................................   1      1    Mm2
.......................................................uaucacagccagcuuugacgU....................................   2      1    Mm2
.......................................................uaucacagccagcuuugaAga....................................   1      1    Mm2
.......................................................uaucacagccagcuuugGcga....................................   1      1    Mm2
.......................................................uaucacagccagcuuugacgG....................................   5      1    Mm2
.......................................................uaucacagccagcuuugacga....................................   204    0    Mm2
.......................................................uaucacagccagcuuuCacga....................................   1      1    Mm2
.......................................................uaucacagccagcuuugGcgag...................................   1      1    Mm2
.......................................................uaucacagcUagcuuugacgag...................................   2      1    Mm2
.......................................................uaucacGgccagcuuugacgag...................................   1      1    Mm2
.......................................................uaucacagccagcuuugacgag...................................   235    0    Mm2
.......................................................uaucacagccagcuuugCcgag...................................   1      1    Mm2
.......................................................uaucacagccagcuuuUacgag...................................   1      1    Mm2
.......................................................uaucacagccagcuuugacgaA...................................   2      1    Mm2
.......................................................uaucacagccagcuuuCacgag...................................   2      1    Mm2
.......................................................uaucacagccagcuuugacgaU...................................   9      1    Mm2
.......................................................uaucaUagccagcuuugacgag...................................   1      1    Mm2
.......................................................uaucacaAccagcuuugacgagc..................................   1      1    Mm2
.......................................................uauUacagccagcuuugacgagc..................................   1      1    Mm2
.......................................................uaucacagccagcuuugacgaUc..................................   1      1    Mm2
.......................................................uGucacagccagcuuugacgagc..................................   2      1    Mm2
.......................................................uaucacagccagcAuugacgagc..................................   1      1    Mm2
.......................................................Naucacagccagcuuugacgagc..................................   2      1    Mm2
.......................................................uaucacagcUagcuuugacgagc..................................   3      1    Mm2
```

Star **Mature**

```
gaaguuggaugcccccagagcagucaaaguggcugugauguguuguucaaaaucauaucacagccagcuuugacgagcuuuguggguguugucgcuuucauugcguaaggca
.......................................................uaucacagccagcuuugacgagA..................................   56     1   Mm2
.......................................................uaucacagccagcuuugacgagU..................................   130    1   Mm2
.......................................................uaucacagccGgcuuugacgagc..................................   9      1   Mm2
.......................................................uaucacagccagcuuugacgaAc..................................   1      1   Mm2
.......................................................uaucacagccagcuuugacgagG..................................   2      1   Mm2
.......................................................uaucacagccagcuuugacgUgc..................................   1      1   Mm2
.......................................................uCucacagccagcuuugacgagc..................................   1      1   Mm2
.......................................................uaCcacagccagcuuugacgagc..................................   2      1   Mm2
.......................................................uaucacagccagcuuugUcgagc..................................   8      1   Mm2
.......................................................uaAcacagccagcuuugacgagc..................................   3      1   Mm2
.......................................................uaucacagccagUuuugacgagc..................................   2      1   Mm2
.......................................................uaucacagccUgcuuugacgagc..................................   14     1   Mm2
.......................................................Caucacagccagcuuugacgagc..................................   2      1   Mm2
.......................................................uaucacagccagcuuuCacgagc..................................   8      1   Mm2
.......................................................uUucacagccagcuuugacgagc..................................   1      1   Mm2
.......................................................uaucacagccCgcuuugacgagc..................................   4      1   Mm2
.......................................................uaucacagccagcuuugacgagc..................................   3295   0   Mm2
.......................................................uaucacagcGagcuuugacgagc..................................   1      1   Mm2
.......................................................uaucacagccagcuuuAacgagc..................................   6      1   Mm2
.......................................................uaucacagccagcuuugacCagc..................................   1      1   Mm2
.......................................................uaucacagccagcuuuUacgagc..................................   6      1   Mm2
.......................................................uaucacagccagcuuugCcgagc..................................   7      1   Mm2
.......................................................uaucacagccagcuuugGcgagc..................................   7      1   Mm2
.......................................................uaucacUgccagcuuugacgagc..................................   2      1   Mm2
.......................................................uaucacagccaUcuuugacgagc..................................   1      1   Mm2
.......................................................uaucacagccUgcuuugacgagcu.................................   3      1   Mm2
.......................................................uaucacagccGgcuuugacgagcu.................................   3      1   Mm2
.......................................................uaucacagccagcuuugGcgagcu.................................   3      1   Mm2
.......................................................uaucacagcUagcuuugacgagcu.................................   2      1   Mm2
.......................................................uaucacaUccagcuuugacgagcu.................................   1      1   Mm2
.......................................................uaucacagccagUuuugacgagcu.................................   1      1   Mm2
.......................................................uaucaUagccagcuuugacgagcu.................................   1      1   Mm2
.......................................................uaucacagccagcuuugacgaAcu.................................   1      1   Mm2
.......................................................uaucacagccagcuuuCacgagcu.................................   4      1   Mm2
.......................................................uaucacagccagcuuugacgagcu.................................   1872   0   Mm2
.......................................................uaucacagccagcuuCgacgagcu.................................   1      1   Mm2
.......................................................uaucacagccagcuuugacgaUcu.................................   2      1   Mm2
.......................................................uaucacagccagcuuugCcgagcu.................................   6      1   Mm2
.......................................................uaucacagccagcuuugacgagcA.................................   12     1   Mm2
.......................................................uaucacagccagcuuugUcgagcu.................................   4      1   Mm2
.......................................................uaucacaAccagcuuugacgagcu.................................   1      1   Mm2
.......................................................uaucacagccagcuuugacUagcu.................................   1      1   Mm2
.......................................................uaucacagccagcCuugacgagcu.................................   1      1   Mm2
.......................................................uauGacagccagcuuugacgagcu.................................   1      1   Mm2
.......................................................uaucCcagccagcuuugacgagcu.................................   1      1   Mm2
.......................................................uaucacagccagcuuuUacgagcu.................................   3      1   Mm2
.......................................................uaucacagccagcuuuAacgagcu.................................   5      1   Mm2
.......................................................uaucacagccagcuuugacgagcG.................................   78     1   Mm2
.......................................................uaucacagcAagcuuugacgagcu.................................   1      1   Mm2
.......................................................uaucacagccCgcuuugacgagcu.................................   1      1   Mm2
.......................................................uauAacagccagcuuugacgagcu.................................   2      1   Mm2
.......................................................uaucacagUcagcuuugacgagcu.................................   1      1   Mm2
.......................................................uCucacagccagcuuugacgagcu.................................   1      1   Mm2
.......................................................uaucacagccagcuuugacgagcC.................................   2      1   Mm2
.......................................................uaucacagccagcuGugacgagcu.................................   1      1   Mm2
.......................................................uGucacagccagcuuugacgagcu.................................   2      1   Mm2
.......................................................uaucacagccagcuuugacgagUu.................................   4      1   Mm2
.......................................................uUucacagccagcuuugacgagcu.................................   1      1   Mm2
.......................................................uaucacagccagcuuugacgagAu.................................   1      1   Mm2
.......................................................uaucacagccagAuuugacgagcu.................................   1      1   Mm2
.......................................................uaucacagccagcuuugacgagcuA................................   8      1   Mm2
.......................................................uaucacagccagcuuugacgagAuu................................   1      1   Mm2
.......................................................uaucacagccagcuuugacgagcuu................................   53     0   Mm2
.......................................................uaucacagccagcuuugUcgagcuu................................   1      1   Mm2
.......................................................uaucacagccagcuuugaUgagcuuu...............................   7      1   Mm2
.......................................................uaucacagccagcuuugacgagcuuu...............................   7      0   Mm2
.......................................................uaucacagccagcuuugacgagcuuuU..............................   1      1   Mm2
........................................................aucacagccagcuuugacgagA..................................   2      1   Mm2
........................................................aucacagccagcuuugacgagc..................................   2      0   Mm2
........................................................aucacagccagcuuugacgagcu.................................   1      0   Mm2
```

Star **Mature**

```
gaaguuggaugcccccagagcagucaaaguggcugugauguguuguucaaaaucauaucacagccagcuuugacgagcuuuguggguguugucgcuuucauugcguaaggca
.........................................................ucacagccagcuuugacgagc..................................  6     0  Mm2
.........................................................ucacagccagcuuugaGgagc..................................  5     1  Mm2
.........................................................ucacagccagcuuugaAgagc..................................  1     1  Mm2
.........................................................ucacagccagcuuugacgagcC.................................  9     1  Mm2
.........................................................ucacagccagcuuugacgagcCu................................  5     1  Mm2

....................cagucaaaguggcugugaugug......................................................................  26    0  Mm1
.......................................................uaucacagccagcuuuga.......................................  7     0  Mm1
.......................................................uaucacagccagcuuugU.......................................  3     1  Mm1
.......................................................uaucacagccagcuuugac......................................  10    0  Mm1
.......................................................uaucacagccagcuuugacg.....................................  14    0  Mm1
.......................................................uaucacagccagcuuugacgG....................................  1     1  Mm1
.......................................................uaucacagccagcuuuCacga....................................  1     1  Mm1
.......................................................uaucacagccagcuuugacga....................................  26    0  Mm1
.......................................................uaucacagccGgcuuugacgag...................................  1     1  Mm1
.......................................................uaucacagccCgcuuugacgag...................................  1     1  Mm1
.......................................................uaucacagccagcuuuAacgag...................................  1     1  Mm1
.......................................................uaucacagccagcuuugCcgag...................................  1     1  Mm1
.......................................................uaucacagccagcuuugacgag...................................  161   0  Mm1
.......................................................uaucacagccagcuuugacgaU...................................  5     1  Mm1
.......................................................uaucacagccagcuuugGcgagc..................................  3     1  Mm1
.......................................................uaucacagccUgcuuugacgagc..................................  5     1  Mm1
.......................................................Caucacagccagcuuugacgagc..................................  1     1  Mm1
.......................................................uUucacagccagcuuugacgagc..................................  1     1  Mm1
.......................................................uaucacagccGgcuuugacgagc..................................  2     1  Mm1
.......................................................uaucaUagccagcuuugacgagc..................................  1     1  Mm1
.......................................................uaucacagccagcuuugacgagU..................................  42    1  Mm1
.......................................................uaucacagccagcuuugCcgagc..................................  7     1  Mm1
.......................................................uaucacagUcagcuuugacgagc..................................  2     1  Mm1
.......................................................uaucacagccagcuuuUacgagc..................................  6     1  Mm1
.......................................................Naucacagccagcuuugacgagc..................................  1     1  Mm1
.......................................................uaucacagccagcuuuCacgagc..................................  2     1  Mm1
.......................................................uaucacagccagcuuugUcgagc..................................  4     1  Mm1
.......................................................uaucacagccagcuuugacgagc..................................  1466  0  Mm1
.......................................................uaucacagccagcuuugacgaUc..................................  3     1  Mm1
.......................................................uaucacagccagcuuuAacgagc..................................  3     1  Mm1
.......................................................uaucacagccagcuuGgacgagc..................................  1     1  Mm1
.......................................................uaucacagccagcuuugacgagA..................................  10    1  Mm1
.......................................................uaucacagccagcuuugUcgagcu.................................  2     1  Mm1
.......................................................uaucacagccagcuuugCcgagcu.................................  3     1  Mm1
.......................................................uaucacagccagcuuuUacgagcu.................................  1     1  Mm1
.......................................................uaucacagccagcuuugacgagcu.................................  845   0  Mm1
.......................................................uaucacagccagcuuuAacgagcu.................................  2     1  Mm1
.......................................................uaucaUagccagcuuugacgagcu.................................  3     1  Mm1
.......................................................uaucacagccagcuuugacgagcG.................................  1     1  Mm1
.......................................................uaucacagccCgcuuugacgagcu.................................  1     1  Mm1
.......................................................uaucacGgccagcuuugacgagcu.................................  1     1  Mm1
.......................................................uaucacagccagcuuuCacgagcu.................................  2     1  Mm1
.......................................................uaucacagccGgcuuugacgagcu.................................  1     1  Mm1
.......................................................Naucacagccagcuuugacgagcu.................................  3     1  Mm1
.......................................................uaucacagccagcuuugGcgagcu.................................  2     1  Mm1
.......................................................uaucacagccagcuuugacgagcA.................................  7     1  Mm1
.......................................................uaAcacagccagcuuugacgagcu.................................  3     1  Mm1
.......................................................uaucacagccagcuuugacgagcC.................................  1     1  Mm1
.......................................................uaucacagccagcuuugacgagcuu................................  12    0  Mm1
.......................................................uaucacagccagcuuugacgagcuC................................  5     1  Mm1
.......................................................uaucacagccagcuuugacgagcuAu...............................  3     1  Mm1
........................................................aucacagccagcuuugacgUgc..................................  1     1  Mm1
........................................................aucacagccagcuuugacgagc..................................  1     0  Mm1
.........................................................ucacagccagcuuugaGgagc..................................  1     1  Mm1
.........................................................ucacagccagcuuugacgagcC.................................  1     1  Mm1
.........................................................ucacagccagcuuugacgagcCu................................  1     1  Mm1
.........................................................ucacagccagcuuugacgagcCuu...............................  1     1  Mm1
...............................................................................uuguggguguugucgcuuuU.............  7     1  Mm1

.......................................................uaucacagccagcuuuga.......................................  1     0  Fm2
.......................................................uaucacagccagcuuugacg.....................................  35    0  Fm2
.......................................................uaucacagccagcuuugUcg.....................................  1     1  Fm2
.......................................................uaAcacagccagcuuugacga....................................  1     1  Fm2
.......................................................uaucacagccagcuuugacgG....................................  5     1  Fm2
```

```
                    Star                        Mature
gaaguuggaugcccccagagcagucaaaguggcugugauguguuguucaaaaucauaucacagccagcuuugacgagcuuugugggguguugucgcuuucauugcguaaggca
.......................................................uaucacagccagcuuugacga.....................................    163    0    Fm2
.......................................................uaucacagccagcuuugUcga.....................................    1      1    Fm2
.......................................................uaucacagccagUuuugacga.....................................    4      1    Fm2
.......................................................uaucacagccagcuuugacgag....................................    147    0    Fm2
.......................................................uaucacagccagcuuugacgaU....................................    17     1    Fm2
.......................................................uaucacagccCgcuuugacgag....................................    1      1    Fm2
.......................................................uaucacagccagcuuuCacgag....................................    1      1    Fm2
.......................................................uaucacagccagcuuugacgGg....................................    1      1    Fm2
.......................................................uaucacagUcagcuuugacgagc...................................    6      1    Fm2
.......................................................uaucacagccGgcuuugacgagc...................................    6      1    Fm2
.......................................................uaucacagcAagcuuugacgagc...................................    2      1    Fm2
.......................................................uaucacagccagcuuugacgaUc...................................    1      1    Fm2
.......................................................uaucacagccagAuuugacgagc...................................    1      1    Fm2
.......................................................uaucacagccagcuuugUcgagc...................................    19     1    Fm2
.......................................................uaucGcagccagcuuugacgagc...................................    1      1    Fm2
.......................................................uaucacagccCgcuuugacgagc...................................    8      1    Fm2
.......................................................uaucaAagccagcuuugacgagc...................................    1      1    Fm2
.......................................................uaucacagccagcuuCgacgagc...................................    3      1    Fm2
.......................................................uaucacGgccagcuuugacgagc...................................    2      1    Fm2
.......................................................uaucacagccagcuuugacUagc...................................    2      1    Fm2
.......................................................uaucacagccagcuuuAacgagc...................................    13     1    Fm2
.......................................................uaucacagcGagcuuugacgagc...................................    1      1    Fm2
.......................................................uaucacagccagcuuugGcgagc...................................    13     1    Fm2
.......................................................uaucacagccagcuuuCacgagc...................................    20     1    Fm2
.......................................................uUucacagccagcuuugacgagc...................................    1      1    Fm2
.......................................................uaucacagccaAcuuugacgagc...................................    1      1    Fm2
.......................................................uaucacagccagcuuugacgagG...................................    5      1    Fm2
.......................................................uaucUcagccagcuuugacgagc...................................    2      1    Fm2
.......................................................uaucacagccagcuuugacgagU...................................    156    1    Fm2
.......................................................Caucacagccagcuuugacgagc...................................    1      1    Fm2
.......................................................uauGacagccagcuuugacgagc...................................    1      1    Fm2
.......................................................uaucacagccUgcuuugacgagc...................................    17     1    Fm2
.......................................................uaGcacagccagcuuugacgagc...................................    3      1    Fm2
.......................................................uaAcacagccagcuuugacgagc...................................    5      1    Fm2
.......................................................uaucacagccagUuuugacgagc...................................    11     1    Fm2
.......................................................uaucacagccagcuuugacgagA...................................    47     1    Fm2
.......................................................uauUacagccagcuuugacgagc...................................    1      1    Fm2
.......................................................uaucacagccagcCuugacgagc...................................    6      1    Fm2
.......................................................uaucacagccagcuuuUacgagc...................................    10     1    Fm2
.......................................................Naucacagccagcuuugacgagc...................................    3      1    Fm2
.......................................................uaucacagccagcAuugacgagc...................................    1      1    Fm2
.......................................................uaucaUagccagcuuugacgagc...................................    2      1    Fm2
.......................................................uaucacagccagcuuugacAagc...................................    3      1    Fm2
.......................................................uCucacagccagcuuugacgagc...................................    1      1    Fm2
.......................................................uGucacagccagcuuugacgagc...................................    3      1    Fm2
.......................................................uaucaNagccagcuuugacgagc...................................    3      1    Fm2
.......................................................uaucacaUccagcuuugacgagc...................................    2      1    Fm2
.......................................................uaucacagcUagcuuugacgagc...................................    2      1    Fm2
.......................................................uaucacagccagcuuugacgagc...................................    5662   0    Fm2
.......................................................uaucacagccagcuuAgacgagc...................................    1      1    Fm2
.......................................................uaucacagAcagcuuugacgagc...................................    1      1    Fm2
.......................................................Aaucacagccagcuuugacgagc...................................    1      1    Fm2
.......................................................uaucacagccagcuuugCcgagc...................................    16     1    Fm2
.......................................................uaucacagccagcGuugacgagc...................................    1      1    Fm2
.......................................................uauAacagccagcuuugacgagc...................................    2      1    Fm2
.......................................................uaucacagccagcuuugUcgagcu..................................    6      1    Fm2
.......................................................uaucacagccagGuuugacgagcu..................................    1      1    Fm2
.......................................................uaucacagccagcuuuUacgagcu..................................    2      1    Fm2
.......................................................uaucacagccagcuuuAacgagcu..................................    1      1    Fm2
.......................................................uaucacagccagcuuugacgagAu..................................    13     1    Fm2
.......................................................uaucacCgccagcuuugacgagcu..................................    1      1    Fm2
.......................................................uaucGcagccagcuuugacgagcu..................................    1      1    Fm2
.......................................................uaucacagccagcuuugacgagUu..................................    15     1    Fm2
.......................................................uaucacagccGgcuuugacgagcu..................................    1      1    Fm2
.......................................................uaucacagccagcuuugacgagcG..................................    82     1    Fm2
.......................................................uaucaUagccagcuuugacgagcu..................................    1      1    Fm2
.......................................................uaucaNagccagcuuugacgagcu..................................    1      1    Fm2
.......................................................uaucacaUccagcuuugacgagcu..................................    1      1    Fm2
.......................................................uaucacagcUagcuuugacgagcu..................................    2      1    Fm2
.......................................................uaucacagcAagcuuugacgagcu..................................    1      1    Fm2
```

Star Mature

```
gaaguuggaugcccccagagcagucaaaguggcugugauguguuguucaaaaucauaucacagccagcuuugacgagcuuuguggggguguugucgcuuucauugcguaaggca
......................................................uaucacagccagcuuuCacgagcu...................................  1     1  Fm2
......................................................uCucacagccagcuuugacgagcu...................................  1     1  Fm2
......................................................uauAacagccagcuuugacgagcu...................................  1     1  Fm2
......................................................Aaucacagccagcuuugacgagcu...................................  1     1  Fm2
......................................................uaucacagccagcuuugacAagcu...................................  2     1  Fm2
......................................................uaucacagccCgcuuugacgagcu...................................  2     1  Fm2
......................................................uaucacagccagcuuugGcgagcu...................................  3     1  Fm2
......................................................uaucacagUcagcuuugacgagcu...................................  1     1  Fm2
......................................................uaucacagccagcuuugacgagcu...................................  1190  0  Fm2
......................................................uaucacagccagcuuugCcgagcu...................................  3     1  Fm2
......................................................uaucacagccagcuuugacgagcuu..................................  10    0  Fm2
......................................................uaucacagccagcuuugaUgagcuuu.................................  8     1  Fm2
........................................................ucacagccagcuuugacgagc....................................  2     0  Fm2
........................................................ucacagccagcuuugaGgagc....................................  1     1  Fm2
........................................................ucacagccagcuuugacgagcC...................................  14    1  Fm2
........................................................ucacagccagcuuugacgagcCu..................................  1     1  Fm2
........................................................ucacagccagcuuugacgagcCuu.................................  1     1  Fm2

....................cagucaaaguggcugugaugug.......................................................................  9     0  Fm1
......................................................uaucacagccagcuuuga.........................................  7     0  Fm1
......................................................uaucacagccagcuuugU.........................................  1     1  Fm1
......................................................uaucacagccagcuuugaA........................................  6     1  Fm1
......................................................uaucacagccagcuuugUc........................................  1     1  Fm1
......................................................uaucacagccagcuuugac........................................  7     0  Fm1
......................................................uaucacagccagcuuugacA.......................................  4     1  Fm1
......................................................uaucacagccagcuuugaGg.......................................  1     1  Fm1
......................................................uaucacagccagcuuugacg.......................................  43    0  Fm1
......................................................uaucacagccagcuuugacU.......................................  19    1  Fm1
......................................................uaucacagccagcuuuCacga......................................  1     1  Fm1
......................................................uaucacagcUagcuuugacga......................................  1     1  Fm1
......................................................uaucacagccGgcuuugacga......................................  2     1  Fm1
......................................................uaucCcagccagcuuugacga......................................  1     1  Fm1
......................................................uaucacagccagcuuugacgC......................................  2     1  Fm1
......................................................uaucacagccagcuuugacgU......................................  5     1  Fm1
......................................................uaCcacagccagcuuugacga......................................  1     1  Fm1
......................................................uaucacagccagcuuugCcga......................................  4     1  Fm1
......................................................uaucacagccagcuuugacCa......................................  1     1  Fm1
......................................................uaucacagccagcuuugacgG......................................  17    1  Fm1
......................................................uaucacagccagcuuugUcga......................................  5     1  Fm1
......................................................uaucacagccagcuuugGcga......................................  3     1  Fm1
......................................................uaucacagccagcuuugacga......................................  789   0  Fm1
......................................................uaucacagccagcuuugacgaU.....................................  91    1  Fm1
......................................................uaucacagccagcuuugacgaC.....................................  1     1  Fm1
......................................................uaucacagccagcuuugacgaA.....................................  6     1  Fm1
......................................................uaucacagccGgcuuugacgag.....................................  1     1  Fm1
......................................................uaucacagccUgcuuugacgag.....................................  1     1  Fm1
......................................................uaucacagccagcuuugacgag.....................................  171   0  Fm1
......................................................uaucacagUcagcuuugacgagc....................................  5     1  Fm1
......................................................uaucacagccagcuuugacgagA....................................  64    1  Fm1
......................................................uaucGcagccagcuuugacgagc....................................  1     1  Fm1
......................................................uaucacagccagcuAugacgagc....................................  1     1  Fm1
......................................................uaucacagccagcuCugacgagc....................................  1     1  Fm1
......................................................uaucacagccagcuuugaAgagc....................................  2     1  Fm1
......................................................uaucaNagccagcuuugacgagc....................................  1     1  Fm1
......................................................uaucacagccagcuuugUcgagc....................................  9     1  Fm1
......................................................uaucacagccagcuuugacUagc....................................  2     1  Fm1
......................................................uaucaUagccagcuuugacgagc....................................  1     1  Fm1
......................................................uaucaAagccagcuuugacgagc....................................  1     1  Fm1
......................................................uaucacagccUgcuuugacgagc....................................  12    1  Fm1
......................................................uaucacagccGgcuuugacgagc....................................  4     1  Fm1
......................................................uaucacagccagcuuuCacgagc....................................  13    1  Fm1
......................................................uGucacagccagcuuugacgagc....................................  1     1  Fm1
......................................................uaucacagccagcuuugacgagG....................................  2     1  Fm1
......................................................uauGacagccagcuuugacgagc....................................  1     1  Fm1
......................................................uaucacagccagUuuugacgagc....................................  6     1  Fm1
......................................................uaucacagccagcuuugacAagc....................................  1     1  Fm1
......................................................uaucacagccagcuuuUacgagc....................................  8     1  Fm1
......................................................uauAacagccagcuuugacgagc....................................  1     1  Fm1
......................................................Naucacagccagcuuugacgagc....................................  2     1  Fm1
......................................................uUucacagccagcuuugacgagc....................................  1     1  Fm1
```

Star **Mature**

```
gaaguuggaugcccccagagcagucaaaguggcugugauguguuguucaaaaucauaucacagccagcuuugacgagcuuuguggguguugucgcuuucauugcguaaggca
.......................................................uauUacagccagcuuugacgagc..................................   1      1   Fm1
.......................................................uaucacagccagcuuuAacgagc..................................   10     1   Fm1
.......................................................uaucacagccagcuuugacgaUc..................................   2      1   Fm1
.......................................................uaucacagccaCcuuugacgagc..................................   1      1   Fm1
.......................................................uaucacagccagcuuugGcgagc..................................   11     1   Fm1
.......................................................uaucacagccagcuuugacgGgc..................................   1      1   Fm1
.......................................................uaucacagcUagcuuugacgagc..................................   8      1   Fm1
.......................................................uaucacagccCgcuuugacgagc..................................   2      1   Fm1
.......................................................uaucacagccagcuuugacgagU..................................   84     1   Fm1
.......................................................uaucacagccagcuuugacgagc..................................   3055   0   Fm1
.......................................................uaucacagccagcuuCgacgagc..................................   1      1   Fm1
.......................................................uaucacagcGagcuuugacgagc..................................   1      1   Fm1
.......................................................uaucacagccagcCuugacgagc..................................   2      1   Fm1
.......................................................uaucacagccagcuuugCcgagc..................................   10     1   Fm1
.......................................................uaucCcagccagcuuugacgagc..................................   1      1   Fm1
.......................................................uaucacagccagcuuugacgagcG.................................   87     1   Fm1
.......................................................uaucaUagccagcuuugacgagcu.................................   1      1   Fm1
.......................................................uaucacagccagcuuugCcgagcu.................................   7      1   Fm1
.......................................................uaucacagccagcuuugacgaAcu.................................   1      1   Fm1
.......................................................uaucacagccagcuuugacgagcA.................................   9      1   Fm1
.......................................................uaucacagccGgcuuugacgagcu.................................   2      1   Fm1
.......................................................uaGcacagccagcuuugacgagcu.................................   1      1   Fm1
.......................................................uaucacagccagGuuugacgagcu.................................   1      1   Fm1
.......................................................uaucacagccagcuGugacgagcu.................................   1      1   Fm1
.......................................................uaucacagccagUuuugacgagcu.................................   1      1   Fm1
.......................................................uaucacagccagcuuugacgagUu.................................   32     1   Fm1
.......................................................uaucacagccagcuuuUacgagcu.................................   3      1   Fm1
.......................................................uGucacagccagcuuugacgagcu.................................   1      1   Fm1
.......................................................uaucacagccagcuuugGcgagcu.................................   4      1   Fm1
.......................................................uaucacagccagcuuugacgUgcu.................................   1      1   Fm1
.......................................................uaucacagccagcuuugacgagcu.................................   1613   0   Fm1
.......................................................uaucacagccUgcuuugacgagcu.................................   3      1   Fm1
.......................................................uaucacagccagcuuuCacgagcu.................................   3      1   Fm1
.......................................................uaucacagcUagcuuugacgagcu.................................   2      1   Fm1
.......................................................uaucacagccagcuuugUcgagcu.................................   1      1   Fm1
.......................................................uaucacagccGgcuuugacgagcuu................................   1      1   Fm1
.......................................................uaucacagccagcuuugacgagcuu................................   43     0   Fm1
.......................................................uaucacagccagcuuugacgagcCu................................   1      1   Fm1
.......................................................uaucacagccagcuuugaUgagcuuu...............................   5      1   Fm1
.......................................................uaucacagccagcuuugacgagcuuu...............................   2      0   Fm1
........................................................aucacagccagcuuugacgagc..................................   4      0   Fm1
........................................................aucacagccagcuuugacgagcu.................................   2      0   Fm1
.........................................................ucacagccagcuuugaGgag...................................   1      1   Fm1
.........................................................ucacagccagcuuugaGgagc..................................   9      1   Fm1
.........................................................ucacagccagcuuuUacgagc..................................   1      1   Fm1
.........................................................ucacagccagcuuugaAgagc..................................   9      1   Fm1
.........................................................ucacagccagcuuugacgagc..................................   11     0   Fm1
.........................................................ucacagccagcuuugacgagcC.................................   8      1   Fm1
.........................................................ucacagccagcuuugacgagcCu................................   1      1   Fm1
..............................................................cagccagcuuugacgagc..................................   5      0   Fm1
..............................................................cagccagcuuugacgagcu.................................   3      0   Fm1
```
